# A Complete Active Space Self-Consistent Field Approach for Molecules in QED Environments

**DOI:** 10.1021/acs.jctc.5c00519

**Published:** 2025-07-07

**Authors:** Riccardo Alessandro, Matteo Castagnola, Henrik Koch, Enrico Ronca

**Affiliations:** † Dipartimento di Chimica, Biologia e Biotecnologie, Università Degli Studi di Perugia, Via Elce di Sotto, 8, 06123 Perugia, Italy; ‡ Department of Chemistry, 8018 Norwegian University of Science and Technology, 7491 Trondheim, Norway

## Abstract

Multireference systems are usually challenging to investigate using ab initio methods as they require an accurate description of static electron correlation. The urgency of developing similar approaches is even more pressing when molecules strongly interact with light in quantum-electrodynamics (QED) environments. In fact, in this context, multireference effects might be induced or reduced by the presence of the field. In this work, we extend the complete active space self-consistent field (CASSCF) approach to polaritonic systems. The method is tested on benchmark multireference problems and applied to investigate field-induced effects on the electronic structure of well-known multiconfigurational processes. Strengths and limitations of the method have been thoroughly analyzed.

## Graphical abstract

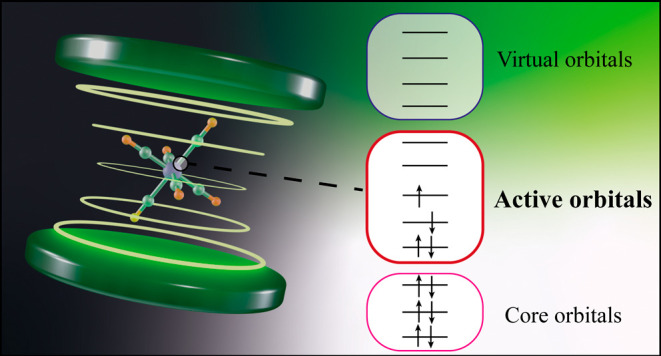

## 1 Introduction

Manipulation of molecular properties by quantum fields is becoming nowadays a very promising nonintrusive alternative to chemical control.^1–8^ The observation of these effects was possible thanks to the significant technological advances made in the fabrication of efficient optical devices (optical,^9,10^ plasmonic^11,12^ and superconducting^13–15^ cavities, waveguides,^16–18^ etc.). The simplest of these devices is the Fabry–Pérot optical cavity (see Figure 1), a device composed of two mirrors whose separation, *L*, is related to the fundamental wavelength of the field inside the cavity. In these conditions, energy is coherently exchanged between the molecule and the cavity field, inducing a phenomenon called Rabi oscillations. Due to the strong light-matter interaction, photons and molecular states lose their individuality and show mixed molecular and photonic character, generating hybrid states known as “polaritons”.^19–22^ The formation of polaritons is associated with a splitting of the energy levels, known as *Rabi splitting* (Ω_R_), that increases with the strength of the light-matter coupling (λ). It is noteworthy to point out that molecules can interact strongly with the field also in the absence of real photons, through a direct coupling between the molecular states and the vacuum fluctuations of the electromagnetic field. In the past decade, a lot of research was devoted to exploring the potential of strong light-matter interaction on a plethora of physical or chemical phenomena. In particular, by coupling molecules or materials with different optical devices, it was possible to manipulate the absorption, emission, and scattering properties of molecules and materials,^23–26^ to induce Bose–Einstein condensation of polaritons,^27–29^ to increase conductivity in organic semiconductors,^30^ or to manipulate the spin properties of matter.^31–33^ One of the most debated but also interesting aspects was observed in chemistry, where field-induced effects on chemical reactivity were demonstrated to inhibit, catalyze, and even make some reactions selective toward specific products.^34–37^

**Figure 1:**
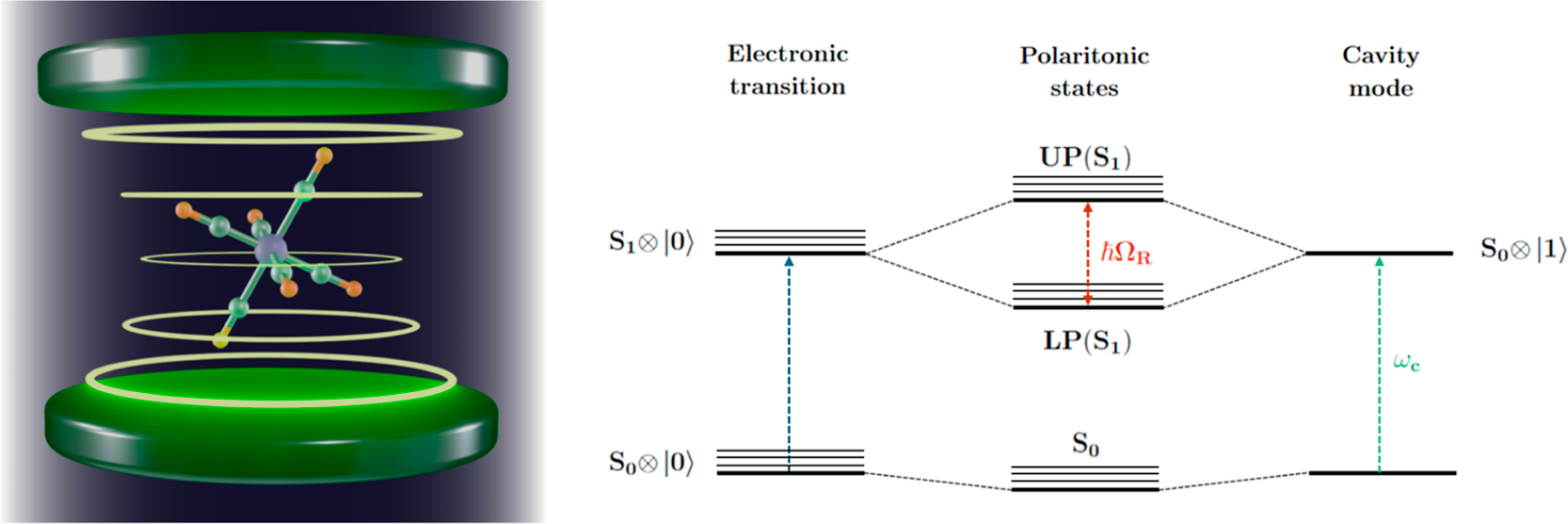
Illustration of a molecule in an optical cavity (left) and energy levels scheme of the polaritonic states obtained by coupling of an electronic transition of a molecule (left) and the cavity field (right). Ω_R_ indicates the Rabi splitting, while ω_c_ is the fundamental electromagnetic field frequency.

Despite the large attention devoted to this field in the past decade, the physics behind many of these processes still remain unclear, and progress is still slow due to the many limitations in the validation of the experimental setup.^38–40^ For these reasons, the development of effective theoretical approaches is crucial to overcome experimental limitations and to propose a consistent description of these phenomena.^41,42^ In recent years, a small number of research groups started developing some extensions to the most commonly used ab initio methodologies, i.e., density functional theory (DFT),^43–45^ Hartree–Fock (HF), full configuration interaction (FCI), coupled cluster (CC),^46–50^ and second-order Møller–Plesset perturbation theory (MP2),^51,52^ to investigate coupled electron-photon systems. Recently, fully relativistic versions of QED-HF and QEDFT have also been proposed to investigate molecular systems containing heavy atoms in strong coupling conditions.^53,54^ The quantum electrodynamics (QED) extensions to HF and DFT are quite efficient and can be easily applied to relatively large systems. However, they completely miss correlation or describe it in a very approximate way. Moreover, in the DFT case, an effective exchange–correlation functional for the electron-photon interaction is still far from being formulated.^41^

Coupled Cluster theory is known to be the most reliable method in quantum chemistry to simulate molecular systems and also for interacting electron-photon systems, its QED extension is at the moment the reference ab initio methodology. However, as for the standard electronic theory, the applicability of this method is limited to small-to medium-sized molecules, and its description becomes qualitatively poor when multiconfigurational effects come into play. This problem is well-known and it prevents the investigation of a relatively large class of complexes, including several systems involved in chemically and biologically interesting processes.^55^ For example, relevant molecular systems that can hardly be described with CC methodologies include open-shell transition metal complexes, (poly)­radicalic systems, excited states of organic molecules, and torsion or breakings of conjugated bonds.^56,57^

The state-of-the-art reference approaches usually used to investigate these systems are the multiconfigurational self-consistent field (MCSCF) methods, which are based on a wave function defined as a linear combination of a reduced number of determinants or configuration state functions (CSFs). A wide variety of MCSCF methods have been developed. The main difference between them lies in the protocol employed to select the determinants to include in the expansion.^58^ The most popular MCSCF method is the complete active space self-consistent field (CASSCF), which is based on a partitioning of the orbital space into three classes: *inactive*, *virtual*, and *active* orbitals. The multideterminantal expansion in the case of CASSCF theory is obtained by choosing a certain number of so-called *active electrons* and computing all the possible excitations of these electrons within the active space. If the active space is chosen properly (i.e., the most correlated orbitals and electrons are included), the CASSCF wave function is able to provide a qualitatively correct description of the multireference molecular system. Despite this method being equivalent to a FCI expansion within the active space, with the current computational facilities, it can be applied, in a relatively efficient way, to active spaces with up to 20 electrons in 20 orbitals.^59^

In the latest years, some of the most common ab initio multireference methods have been extended to coupled light-matter systems.^60–62^ In particular, the implementation of QED-CASCI and QED-DMRG provided the first steps toward the inclusion of static correlation effects in optical cavities. However, the CASCI procedure does not optimize the orbitals, leading to variationally higher energies with respect to its SCF counterpart,^63^ and although the DMRG method has a polynomial scaling, its cost is higher than CASSCF for small active spaces. The comparatively higher computational cost of DMRG for small active spaces provides a rationale for extending and applying the CASSCF method to the QED case.

In this work, we present an extension of the state-specific (SS-) CASSCF theory to coupled light-matter systems (QED-CASSCF). The implementation is performed following a restricted-step second-order optimization scheme, according to the implementations in refs 64–67 . Recently, a QED version of the state-averaged (SA) variant of CASSCF was proposed by Vu et al.^68^ in parallel to this work. It was applied to a small test case with an active space close to the full orbital space, with results very close to the FCI limit. In this work, we perform simulations of larger systems where the CASSCF method does not approach the FCI limit. In this situation, a deep analysis of the method’s performances to describe field-induced effects on multireference systems could be performed.

This paper is organized as follows: in Section 2, a brief summary of the CASSCF method and its second-order implementation is presented. In Section 3, the fundamental details of QED-CASSCF are described. In Section 4, QED-CASSCF is tested on small molecules against other ab initio methodologies. In this section, QED-CASSCF is also used to investigate the field effects on well-known multireference chemical processes. We conclude the work with future perspectives presented in Section 5.

## 2 CASSCF Theory

In MCSCF theory the wave function is written as the linear combination of a certain number of Slater determinants or configuration state functions (CSFs)

1 $$|\Psi^{MCSCF}\rangle =\sum_{I}C_{I}|I\rangle$$

and it is optimized by variationally minimizing the energy with respect to the molecular orbital-rotation parameters and configuration interaction (CI) coefficients (**κ** and ***c***, respectively)

2 $$E=\min_{\kappa ,c}\frac{\left\langle \Psi \left|Ĥ\right|\Psi \right\rangle }{\left\langle \Psi |\Psi \right\rangle }$$

Among the MCSCF methods,^69–73^ the complete active space self-consistent field (CASSCF) approach is still the state-of-the-art procedure for molecular systems dominated by static correlation. The orbital space of a molecule in the CASSCF approximation is partitioned into three subspaces, as depicted in Figure 2: inactive orbitals are always fully occupied, active orbitals have noninteger occupations, and virtual orbitals are always empty. In this work the different orbitals are addressed according to the following indices choice: *i*, *j*, *k*, *l* for inactive orbitals, *u*, *v*, *x*, *y* for active orbitals, *a*, *b*, *c*, *d* for virtual orbitals, and *p*, *q*, *r*, *s* for generic orbitals.

**Figure 2:**
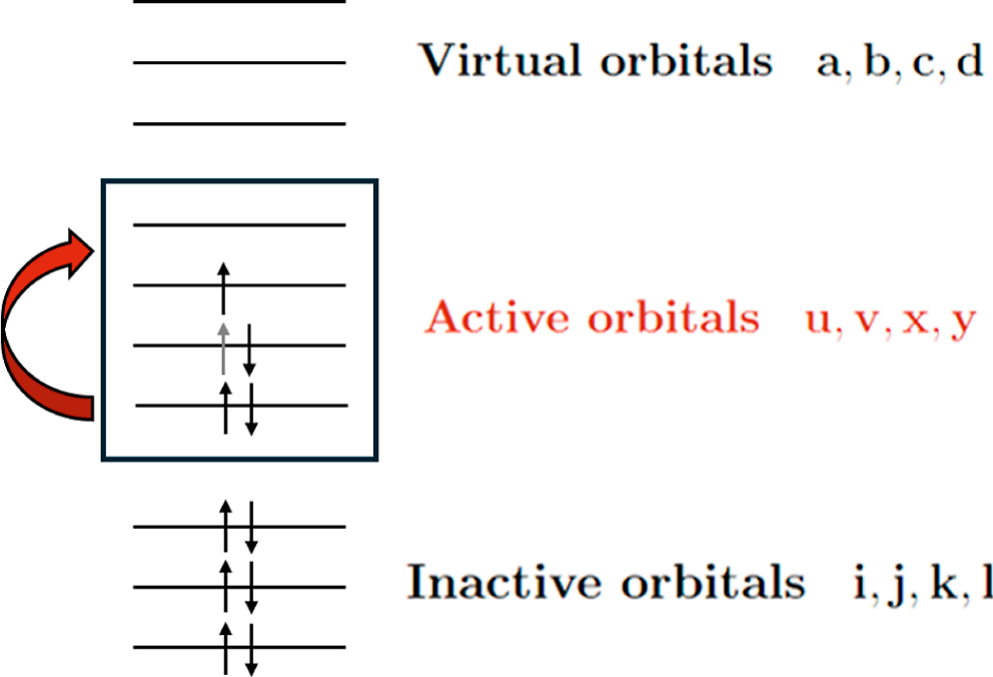
CASSCF orbital partition scheme.

The multideterminantal expansion for the wave function is obtained by considering all the determinants computed by distributing *N* active electrons in *M* active orbitals, namely CAS­(*N*,*M*).

The first implementations of CASSCF relied on first-order solvers, and among these, the super-CI approach is the most widely applied.^74,75^ Although recent first-order implementations proved to be very efficient,^76^ during the optimization of the CASSCF wave function, convergence issues may arise. This led to the development of robust second-order quadratically convergent solvers, which exhibit better convergence properties in a reduced number of iterations at the price of a higher cost for each step.^64,67,77–85^ CASSCF implementations based on second-order algorithms are nowadays relatively well-established in the quantum chemistry community and are available in the majority of the most used software packages.^86–90^ This approach is particularly convenient for the application to simulate small molecular systems, and calculations on larger systems are nowadays efficiently performed with the aid of parallelization, AO-based implementations, or by exploiting the Cholesky decomposition or the resolution of identity (RI) approximation of the two-electron integrals.^67,85^ In MCSCF theory, the first step toward the implementation of the method is an appropriate choice of the parametrization for the wave function. The orbital part is parametrized in terms of an exponential transformation with the anti-Hermitian operator κ̂ defined as

3 $$\mathrm{\kappa ̂}=\sum_{p>q}k_{pq}{Ê}_{pq}^{-}=\sum_{p>q}k_{pq}\left({Ê}_{pq}-{Ê}_{qp}\right)$$

where ${Ê}_{pq}=\sum_{\sigma }{â}_{p\sigma }^{†}{â}_{q\sigma }$ is the spin traced singlet excitation operator with σ ∈ {α, β}. While no alternatives to the parametrization of the orbital space are available, the CI space is liable to multiple choices depending on the CASSCF variant that has to be implemented. In the case of state-specific (SS) CASSCF method, a linear parametrization for the CI part is commonly adopted and the wave function can be written as^64^

4 $$|\Psi \rangle =e^{-\mathrm{\kappa ̂}}\frac{|0\rangle +\mathrm{P̂}|c\rangle }{\sqrt{1+\left\langle c\left|\mathrm{P̂}\right|c\right\rangle }}$$

where |0⟩ is indicated as the *current expansion point* (CEP) and constitutes the current approximation to the wave function. *P̂* = 1 – |0⟩⟨0|, instead, is a projector operator that removes the components parallel to the CEP from the correction vector, |**c**⟩, which contains the variational parameters, *c*_*I*_, with respect to the CI part

5 $$|c\rangle =\sum_{I}c_{I}|I\rangle$$

The choice of the exponential parametrization allows to consider only the nonredundant rotations between the orbital spaces (i.e., orbital rotations that contribute to the energy: inactive–active, inactive-virtual, and active-virtual).^58^ The linear parametrization of the CI space contains only one redundant parameter, that is, when the correction vector |**c**⟩ has components parallel to the reference state |0⟩. However, this is not an issue, as this redundancy can be easily tracked and is projected out by the operator P̂. Therefore, this redundancy does not interfere with the optimization procedure. In the following of this section, we will quickly recap the implementation details of the second-order SS-CASSCF algorithm that will be applied, in the next section, for the extension to strongly coupled electron-photon systems.

### 2.1 Second-Order CASSCF

The implementation of a second-order algorithm is based on the definition of a quadratic model to locally expand the energy up to the second order in **δ**

6 $$E\approx Q\left(\delta \right)=E_{0}+g^{T}\delta +\frac{1}{2}\delta^{T}G\delta$$

where **δ** indicates a small variation of the wave function parameters (**κ**, ***c***). In eq 6, *E*_0_ is the CASSCF energy and **g** is the gradient vector containing the first derivatives of the energy with respect to the configuration (c) and orbital (o) coefficients, all calculated at the current expansion point

7 $$g=\left(\begin{array}{l} g^{c}\\ g^{o} \end{array}\right)=\left(\begin{array}{l} \frac{\partial E}{\partial c}\\ \frac{\partial E}{\partial \kappa } \end{array}\right)$$

Finally, **G** is the Hessian matrix containing the pure configuration (cc), orbital (oo), and the mixed configuration-orbital (co) and orbital-configuration (oc) parts

8 $$G=\left(\begin{array}{ll} G^{cc} & G^{co}\\ G^{oc} & G^{oo} \end{array}\right)=\left(\begin{array}{ll} \frac{\partial^{2}E}{\partial c\partial c} & \frac{\partial^{2}E}{\partial c\partial \kappa }\\ \frac{\partial^{2}E}{\partial \kappa \partial c} & \frac{\partial^{2}E}{\partial \kappa \partial \kappa } \end{array}\right)$$

The Hessian matrix elements are calculated, again, at the current expansion point.

In principle, a second order algorithm requires the explicit computation of the electronic Hessian. However, this procedure can be performed efficiently in a direct fashion. This approach passes through the calculation of the so-called *sigma vectors* defined as

9 $$\sigma =Gb=\left(\begin{array}{ll} G^{cc} & G^{co}\\ G^{oc} & G^{oo} \end{array}\right)\left(\begin{array}{l} b^{c}\\ b^{o} \end{array}\right)=\left(\begin{array}{lll} G^{cc}b^{c} & + & G^{co}b^{o}\\ G^{oc}b^{c} & + & G^{oo}b^{o} \end{array}\right)$$

Here, **b** are the so-called trial vectors used in the Davidson scheme^91^ which contain the wave function parameters with respect to the orbital and configuration parts

10 $$b_{pq}^{o}=\kappa_{pq},,b_{I}^{c}=v_{I}$$

This approach allows for large scale operations without explicitly calculating and storing the blocks of **G** in memory.^92^ Moreover, sigma vectors can be conveniently computed applying minor modifications to intermediate quantities already calculated for the gradients.

By minimizing the quadratic function defined in eq 6 the equation for the Newton step is obtained as

11 Gδ=−g

This procedure is not optimal since it requires the Hessian to be positive-definite. This condition is not always guaranteed, especially at the beginning of the optimization procedure. Even when it is respected, the computed step may not point in the direction of the minimum, leading to a poor convergence.^93^ This problem can be largely reduced by adopting a restricted step procedure.^64,94^ According to this scheme, the choice of the step is restricted within a well-defined *trust region* with radius, *h*

12 $$\delta^{T}\delta \leq h$$

where we assume the potential energy surface to be correctly approximated by the quadratic model. This ensures the Hessian to be positive-definite. This procedure was first described by Levenberg and Marquardt^95,96^ and is based on the solution of the level-shifted Newton equation

13 (G−μI)δ=−g

where the level-shifting parameter μ corresponds to the lowest eigenvalue of the *augmented Hessian*

14 $$L\left(\alpha \right)=\left(\begin{array}{ll} 0 & \alpha g^{T}\\ \alpha g & G \end{array}\right)$$

The relation between the eigenvalues ϵ of **G** and μ of **L**(α) is given by the Hylleraas–Undheim–MacDonald theorem^97,98^

15 $$\mu_{1}\leq \varepsilon_{1}\leq \mu_{2}\leq \cdot \cdot \cdot \leq \mu_{n}\leq \varepsilon_{n}\leq \mu_{n+1}$$

Therefore, the choice of the lowest eigenvalue of the augmented Hessian as a level-shifting parameter guarantees (**G** – μ**I**) to be positive-definite. This approach requires, in principle, to solve the linear system in eq 13 at every iteration. However, the inversion of (**G** – μ**I**) could be computationally inefficient. An alternative way can be obtained by diagonalizing the augmented Hessian in eq 14. This can be done efficiently in an iterative fashion by implementing the sigma vectors in eq 9. Once the lowest eigenvalue and eigenvector are obtained, the step for the new iteration can be computed as

16 $$\delta =\frac{1}{\alpha }x\left(\alpha \right)$$

where **x**(α) is the eigenvector of the Hessian and α is a scaling parameter that forces the step to lie withing the trust region. The trust radius is changed adaptively during the procedure following the strategy described by Fletcher in ref 93. As long as the step is kept within the trust region, the Newton’s step in eq 11 is recovered, and convergence to the closest minimum is guaranteed.

The presented trust-region optimization method can be summarized as follows:

1. compute the gradient in eq 7. If its norm is lower than a certain threshold, convergence is reached;
2. check the step and adjust the trust radius according to the procedure described by Fletcher in ref 93;
3. iteratively diagonalize the augmented Hessian in eq 14;
4. update the wave function parameters (eq 16) and return to step 1.

Details on this procedure are described in refs 58, 64, 78 and 93 . The optimized algorithm as implemented in the $e^{T}$ suite of programs,^99^ is described in ref 66.

## 3 QED-CASSCF

The description of molecular systems in optical cavities requires to account explicitly for the quantum character of the electromagnetic field. In our work, we will use the well-established nonrelativistic single-mode Pauli–Fierz Hamiltonian in length gauge and dipole approximation as a starting point for the development of the QED-CASSCF approach^46^

17 ĤPF=Ĥe+ωb̂†b̂+12(λ·(d̂−⟨d̂⟩))2−ω2(λ·(d̂−⟨d̂⟩))(b̂†+b̂)

here, d̂ is the dipole moment operator, ⟨d̂⟩ is its expectation value, and $\lambda =\sqrt{\frac{4\pi }{V}}e$ is the light-matter coupling parameter that depends on the quantization volume *V* and the polarization vector ***e***. For simplicity, a single cavity mode is included in our treatment, but extension to more modes can be applied in a trivial manner. To ensure origin invariance, the Hamiltonian in eq 17 has already been expressed in the coherent-state basis.^46^ Following the notation used in ref 100, we can conveniently set d̂=d̂·λ and rewrite the Pauli–Fierz Hamiltonian as

18 ĤPF=∑pqhpqÊpq+12∑pqrsgpqrsêpqrs+ωb̂†b̂+ω2∑pqdpqÊpq(b̂†+b̂)−ω2⟨d̂⟩(b̂†+b̂)+ĥnuc

In this form the dipole-self-energy term is included in the one- and two-electron integrals

19 hpq=hpqe+12qpq−dpq⟨d̂⟩+δpq2Ne⟨d̂⟩2gpqrs=gpqrse+dpqdrs

with *d*_*pq*_ and *q*_*pq*_ being the electric dipole and quadrupole integrals, respectively, and *N*_e_ is the number of electrons in the molecule.

For a general QED-MCSCF theory, the ground-state wave function can be expressed as^60^

20 $$|\Psi_{0}\rangle =\sum_{I}\sum_{m}C_{I,m}^{\left(0\right)}|I\rangle ⊗|m\rangle$$

where |*m*⟩ is the *m*th photonic state of the cavity mode. In this case the CI coefficients refer to the noninteracting electron-photon states Φ_*I*,*m*_ defined as

21 $$\Phi_{I,m}=|I\rangle ⊗|m\rangle$$

For the sake of readability of the following section, we conveniently separate the Pauli–Fierz Hamiltonian into three terms

22 $${Ĥ}_{PF}={Ĥ}_{e}+{Ĥ}_{ph}+{Ĥ}_{\mathrm{int}}$$

where we need to remember that *H*_e_ contains the one- and two-electron integrals modified for the dipole self-energy term. From now on, we will refer to the Pauli–Fierz Hamiltonian as *H* unless explicitly stated.

The extension of CASSCF to molecular polaritons and its second-order implementation requires the computation of modified energy contributions, gradients, and sigma vectors. The expression for the QED-CASSCF energy can be written as

23 $$E^{QED‐CASSCF}=\left\langle \Psi_{0}\left|Ĥ\right|\Psi_{0}\right\rangle =E_{el}+E_{ph}+E_{\mathrm{int}}+E_{nuc}$$

where

24 $$E_{el}=\sum_{uv}F_{uv}^{I}\gamma_{uv}+\frac{1}{2}\sum_{uxyv}g_{uvxy}\Gamma_{uvxy}+\sum_{i}\left(h_{ii}+F_{ii}^{I}\right)$$

25 $$\gamma_{uv}=\left\langle \Psi_{0}\left|{Ê}_{uv}\right|\Psi_{0}\right\rangle {,,\Gamma }_{uvxy}=\left\langle \Psi_{0}\left|{ê}_{uvxy}\right|\Psi_{0}\right\rangle$$

Are the one- and two-body reduced density matrices, respectively, where

26 $${ê}_{uvxy}={Ê}_{uv}{Ê}_{xy}-\delta_{xv}{Ê}_{uy}$$

is the two-electron excitation operator running on the active indices.^58^ In eq 24

27 $$F_{pq}^{I}=h_{pq}+\sum_{i}\left(2g_{pqii}-g_{piiq}\right)$$

are the elements of the inactive Fock matrix,^58,77^ and

28 $$E_{i}=\sum_{i}\left(h_{ii}+F_{ii}^{I}\right)$$

is denoted as inactive energy.^85^ The purely photonic contribution to the energy is given by

29 $$E_{ph}=\omega \sum_{I,m}{\left|C_{I,m}^{\left(0\right)}\right|}^{2}m$$

where *m* is the number of photons in the *m*th photonic state of the cavity mode. Finally, the contribution by the bilinear coupling is written as

30 $$E_{\mathrm{int}}=\sqrt{\frac{\omega }{2}}\left\{\left(\sum_{i}2d_{ii}-\left\langle \mathrm{d̂}\right\rangle \right)\sum_{I,m}\left(\sqrt{m+1}C_{I,m+1}^{\left(0\right)}+\sqrt{m}C_{I,m-1}^{\left(0\right)}\right)C_{I,m}^{\left(0\right)}+\sum_{uv}d_{uv}{\mathrm{\gamma ̃}}_{uv}\right\}$$

where the modified one-body density matrix is defined as

31 γ̃uv=∑I,J∑m(m+1CI,m+1(0)⟨I|Êuv|J⟩CJ,m(0)+mCI,m−1(0)⟨I|Êuv|J⟩CJ,m(0))

The general expression for the CI gradient is given by

32 $$g_{I,m}^{c}=2\left\langle \Phi_{I,m}\left|\mathrm{P̂}Ĥ\right|\Psi_{0}\right\rangle =2\left\langle \Phi_{I,m}\left|Ĥ\right|\Psi_{0}\right\rangle -2C_{I,m}^{\left(0\right)}E^{\left(0\right)}$$

where *E* ^(0)^ is the QED-CASSCF energy at the current expansion point. Similarly to the energy, also the CI gradient can be split up into different terms

33 $$g_{I,m}^{c}=2\left(g_{I,m}^{el}+g_{I,m}^{ph}+g_{I,m}^{\mathrm{int}}-C_{I,m}^{\left(0\right)}E^{\left(0\right)}\right)$$

where

34 gI,mel=⟨ΦI,m|Ĥel|Ψ0⟩=EiCI,m(0)+∑J⟨I|∑uvFuvIÊuv+12∑uvxyguvxyêuvxy|J⟩CJ,m(0)

and

35 $$g_{I,m}^{ph}=\left\langle \Phi_{I,m}\left|{Ĥ}_{ph}\right|\Psi_{0}\right\rangle =m\omega C_{I,m}^{\left(0\right)}$$

The bilinear contribution to the CI gradient

36 gI,mint=⟨ΦI,m|Ĥint|Ψ0⟩=ω2∑n(nδm,n−1+n+1δm,n+1){(∑i2dii−⟨d̂⟩)CI,n(0)+∑J∑uvduv⟨I|Êuv|J⟩CJ,n(0)}

The general expression for the orbital part of the gradient from eq 7 is given by

37 $$g_{mn}^{o}=\left\langle \Psi_{0}\left|\left[{Ê}_{mn}^{-},Ĥ\right]\right|\Psi_{0}\right\rangle =2\left\langle \Psi_{0}\left|\left[{Ê}_{mn},Ĥ\right]\right|\Psi_{0}\right\rangle$$

where the purely photonic contribution to the orbital gradient is zero since ${Ê}_{mn}$ and ${Ĥ}_{ph}$ commute. The purely electronic contribution is known^58^

38 $$g_{mn}^{el}=2\left(F_{mn}-F_{nm}\right)$$

where

39 $$F_{mn}=\sum_{q}\gamma_{mq}h_{nq}+\sum_{qrs}\Gamma_{mqrs}g_{nqrs}$$

is the generalized Fock matrix. Finally, the interaction term to the gradient reads as

40 $$g_{pq}^{\mathrm{int}}=2\left\langle \Psi_{0}\left|\left[{Ê}_{pq},{Ĥ}^{\mathrm{int}}\right]\right|\Psi_{0}\right\rangle =2\sqrt{\frac{\omega }{2}}\sum_{r}\left(d_{qr}{\mathrm{\gamma ̃}}_{\mathrm{pr}}-d_{rp}{\mathrm{\gamma ̃}}_{rq}\right)$$

The sigma vectors defined in eq 9 can be calculated as

41a $$\sigma_{I,m}^{cc}=\sum_{J,n}G_{I,m;J,n}^{cc}b_{J,n}^{c}=2\left\langle \Phi_{I,m}\left|\left(Ĥ-E_{0}\right)\right|v\right\rangle -C_{I,m}^{\left(0\right)}\sum_{J,n}g_{J,n}^{c}v_{J,n}-g_{I,m}^{c}\sum_{J,n}C_{J,n}^{\left(0\right)}v_{J,n}$$

41b $$\sigma_{pq}^{oc}=\sum_{I,m}G_{pq;I,m}^{oc}b_{I,m}^{c}=\left\langle v\left|\left[{Ê}_{pq}^{-},Ĥ\right]\right|\Psi_{0}\right\rangle +\left\langle \Psi_{0}\left|\left[{Ê}_{pq}^{-},Ĥ\right]\right|v\right\rangle -2g_{pq}^{o}\sum_{I,m}C_{I,m}^{\left(0\right)}v_{I,m}$$

41c $$\sigma_{I,m}^{co}=\sum_{pq}G_{I,m;pq}^{co}b_{pq}^{o}=2\left\langle \Phi_{I,m}\left|{Ĥ}^{k}\right|\Psi_{0}\right\rangle -2C_{I,m}^{\left(0\right)}\sum_{pq}g_{pq}^{o}\kappa_{rs}$$

41d $$\sigma_{pq}^{oo}=\sum_{rs}G_{pq,rs}b_{rs}^{o}=\left\langle \Psi_{0}\left|\left[{Ê}_{pq}^{-},{Ĥ}^{k}\right]\right|\Psi_{0}\right\rangle +\frac{1}{2}\sum_{s}\left(g_{sp}^{o}\kappa_{qs}-g_{sq}^{o}\kappa_{ps}\right)$$

where we have introduced the modified state

42 $$|v\rangle =\sum_{I}\sum_{m}v_{I,m}|\Phi_{I,m}\rangle$$

${Ĥ}^{k}$ is the one-index transformed Hamiltonian,^58^ where only the terms involving molecular integrals are one-index transformed

43 $${Ĥ}_{el}^{k}=\sum_{pq}h_{pq}^{k}{Ê}_{pq}+\frac{1}{2}\sum_{pqrs}g_{pqrs}^{k}{ê}_{pqrs}$$

44 $${Ĥ}_{\mathrm{int}}^{k}=\sqrt{\frac{\omega }{2}}\sum_{pq}d_{pq}^{k}{Ê}_{pq}\left({\mathrm{b̂}}^{†}+\mathrm{b̂}\right)$$

where

45 $$h_{pq}^{k}=\sum_{m}\left(k_{pm}h_{mq}+k_{qm}h_{pm}\right)$$

46 $$g_{pqrs}^{k}=\sum_{m}\left(k_{pm}g_{mqrs}+k_{qm}g_{pmrs}+k_{rm}g_{pqms}+k_{sm}g_{pqrm}\right)$$

47 $$d_{pq}^{k}=\sum_{m}\left(k_{pm}d_{mq}+k_{qm}d_{pm}\right)$$

Substituting these quantities in eqs 7–9 an efficient second-order algorithm for QED-CASSCF can be naturally obtained.

## 4 Results

In this section, QED-CASSCF will be tested on small multireference systems and used to investigate field-induced multiconfigurational effects. All the calculations are performed with a development version of the $e^{T}$ software package.^99^

### 4.1 Dissociation of the N_2_ Ground State

The homolytic dissociation curve of N_2_ is a typical test case for investigating the behavior of multireference methods. For this system, the active space contains the six valence electrons inside the six p orbitals, i.e., CAS­(6,6). The calculations were performed using a Dunning’s cc-pVDZ basis,^101^ light-matter coupling λ = 0.03 au, cavity frequency ω = 0.5 eV, and the field polarization along the *z* axis, coincident with the main molecular axis. All the QED calculations are performed including a single photonic state. To check the potential effect of a higher number of photons in the photonic Fock space, the calculations were repeated including up to three photonic states, but no relevant difference was observed (see Figure S1 in the Supporting Information). The potential energy curve has been computed over 32 steps from 0.75 to 8.50 Å. In Figure 3a, the potential energy curve computed at the QED-CASSCF level is compared against QED-CASCI and QED-CCSD.

**Figure 3:**
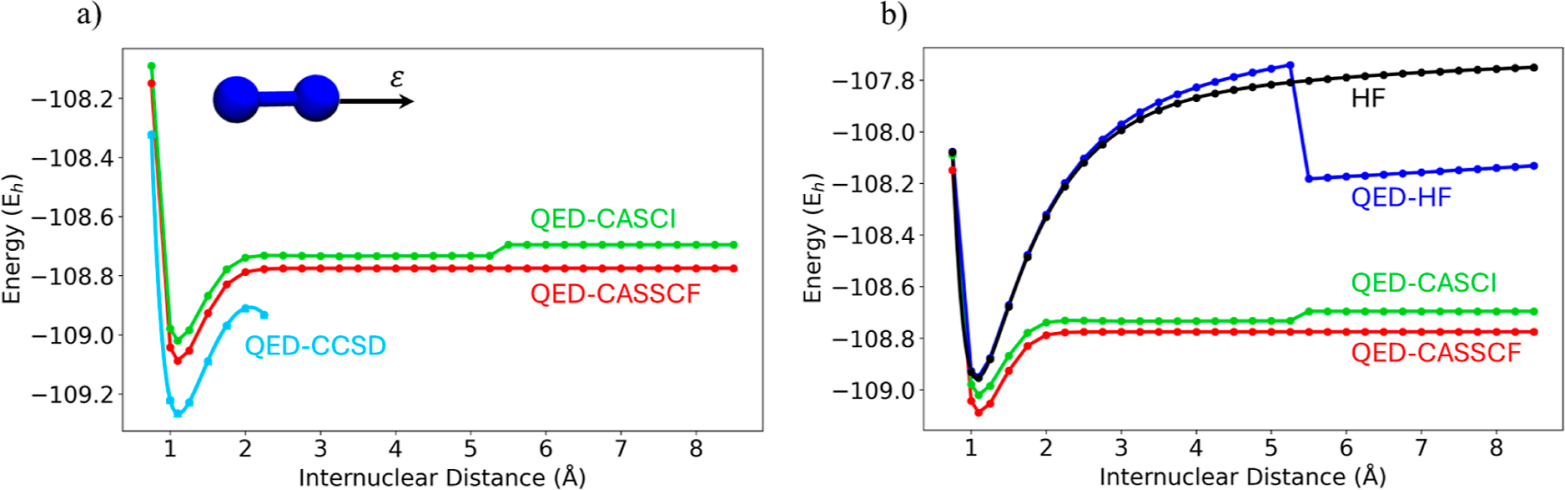
Potential energy curves of the nitrogen molecule along the internuclear distance computed at the (a) QED-CASSCF, QED-CASCI, and QED-CCSD level (b) QED-CASSCF, QED-CASCI, QED-HF, and purely electronic HF.

For distances up to 5 Å, the energy profiles are in qualitative agreement with the purely electronic potential energy curves (PECs), with coupled cluster exhibiting lower energies with respect to CAS-type energies. Notice, however, that, at 2.25 Å, CC, after showing an unphysical maximum around 2.0 Å, stops converging due to the emergence of multireference effects associated with the homolytic dissociation of the system. Despite showing a higher energy for the dissociation plateau compared to both QED-CC and QED-CASSCF, QED-CASCI describes the right energy profile in a wide range of energies, as expected already for the bare electronic system. The higher energy of the dissociated system can be clearly explained by the lack of dynamical correlation (included in QED-CC) and by the use of unoptimized molecular orbitals (present in QED-CASSCF). However, it is important to highlight the presence of an unphysical jump in energy between 5.25 and 5.50 Å which is not registered for the purely electronic profile (see Figure S2 in Supporting Information). This behavior is indeed given by the guess used for the calculation. In fact, while the purely electronic Hartree–Fock potential energy curve is continuous in the whole energy range, the QED-HF curve shows a drastic energy change at the same internuclear distances (see Figure 3b).

The QED-HF orbitals used in Figure 3 are generated independently at every point. To thoroughly understand the unphysical behavior at high distances, we performed the calculations at the QED-HF level to obtain the two full PECs by using different orbitals. A higher-energy curve was obtained by restarting the calculations using the orbitals from smaller internuclear distances while a lower-energy curve was obtained by using the orbitals from larger distances (see Figure 4).

**Figure 4:**
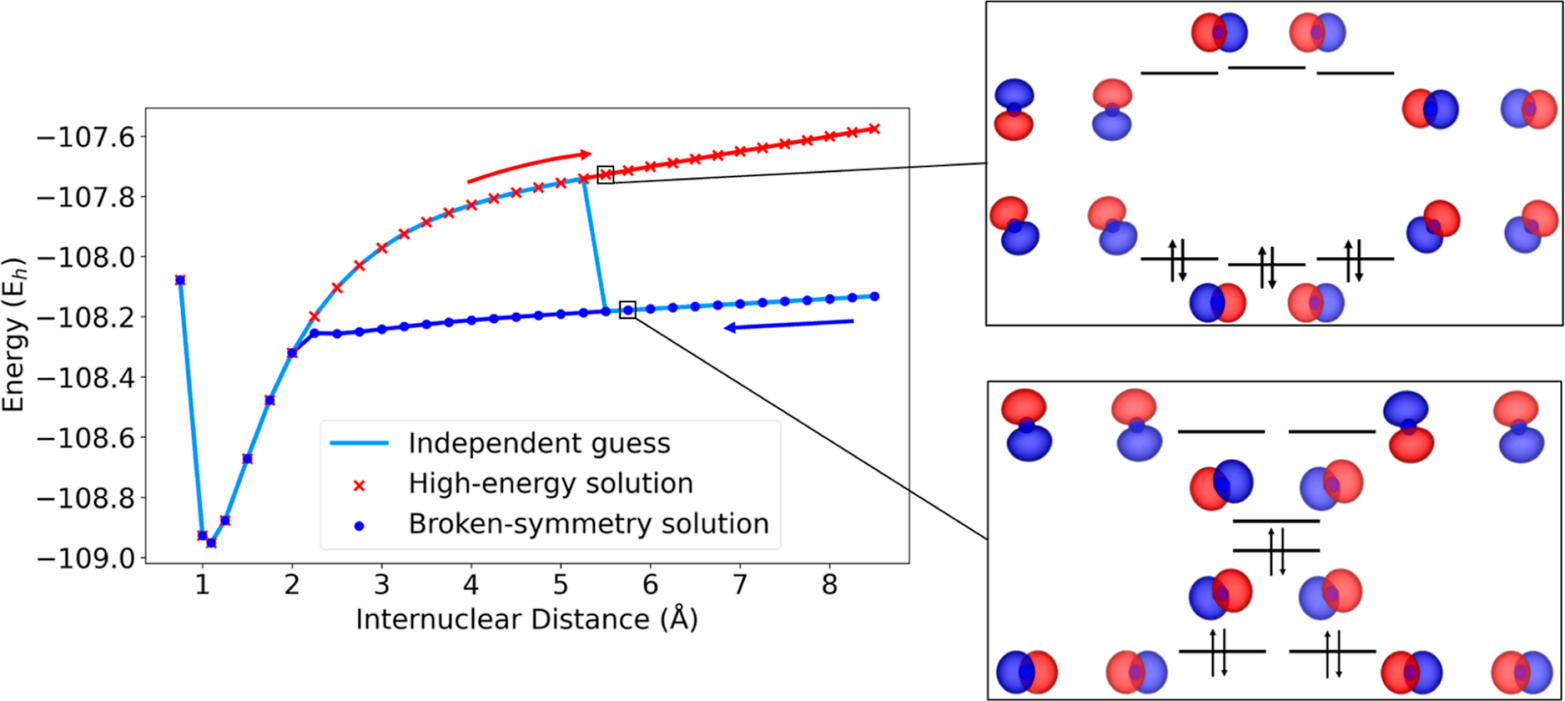
QED-HF potential energy curves for the nitrogen molecule obtained by restarting the calculations using the orbitals from lower to higher distances (in red) and from higher to lower distances (in blue), compared with the curve obtained by generating an independent guess for each point (in light blue).

The higher energy curve reproduces both the profile and the orbital energy ordering of the purely electronic HF dissociation curve while the lower energy curve corresponds to a symmetry broken solution where, as one may expect, the energy ordering of the orbitals is different.

It is possible to use the orbitals from both curves as a guess for QED-CASCI calculations. By doing so, two smooth curves can be obtained, where the one obtained from the higher energy solutions is variationally lower with respect to the other curve (see Figure S3 in Supporting Information). This problem is fixed by QED-CASSCF, as shown in Figure 3. In this case, indeed, the variational optimization of the orbitals avoids discontinuities due to the poor quality of the guess, predicting a continuous energy profile in the whole distance range and, as expected, provides variationally lower energies with respect to the QED-CASCI calculations (see Figure S4 in Supporting Information). This behavior is a demonstration that the presence of the field can, in some cases, complicate the simulation of molecular systems characterized by multireference effects and that the orbital optimization can be important to obtain qualitatively correct results, especially in the cases where multireference effects become dominant.

In order to estimate the entity of the field-induced effects, we also computed the energy difference between the QED-CASSCF method and the corresponding electronic one (see Figure 5) and, as expected, the energy difference goes to a plateau.

**Figure 5:**
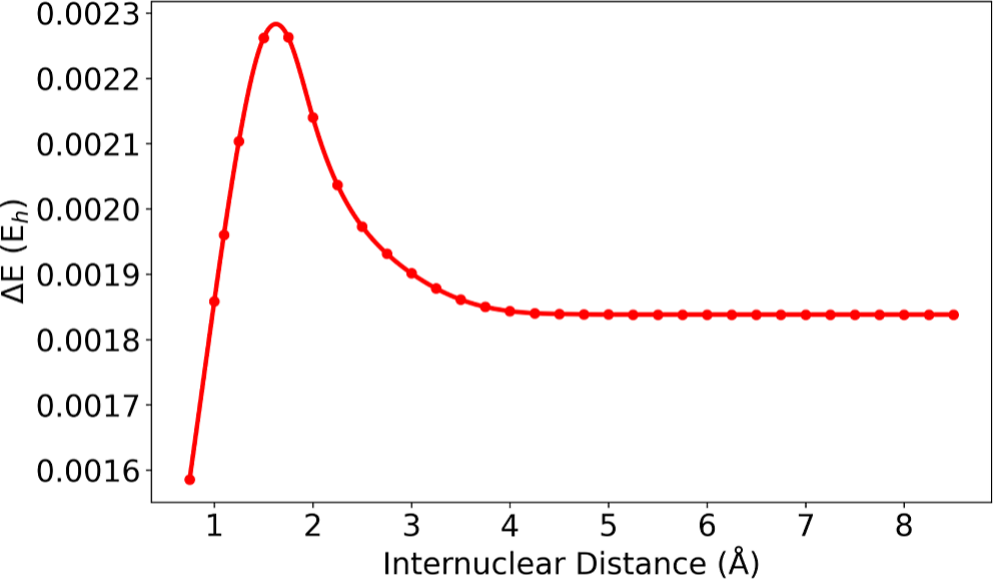
Study of the field-induced effects on the ground state potential energy surfaces computed at the CASSCF level.

**Figure 6:**
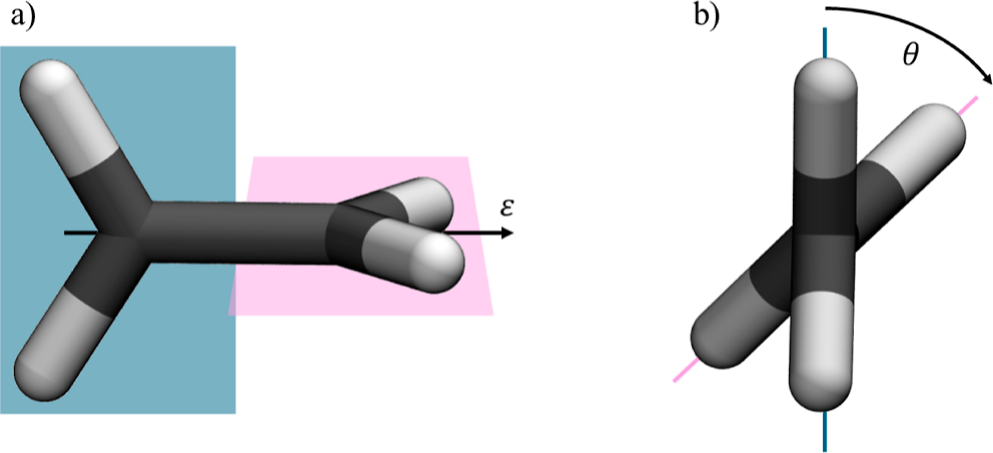
(a) Twisted ethylene molecule, field polarization **ϵ** along the carbon–carbon bond. (b) Representation of the dihedral angle, θ.

Before concluding, it is important to stress that even for very large coupling values (per molecule) like those applied in this test, the field effects on the ground state energy are very small. Much more significant effects could be observed on the excited states energies, but their study goes beyond the scope of this paper and will be analyzed in detail later in a follow-up work.

### 4.2 Field-Induced Effects on the Torsion of Ethylene

The description of bond breakings, torsions along conjugated bonds, and conical intersections has always been a challenge for single-reference methods.^102^ CASSCF is still the most commonly employed method to provide a good guess for the description of such phenomena. In this section we investigate the double bond torsion of an ethylene molecule coupled to an optical cavity (see Figure 6). This molecular system has been largely studied in the literature.^103^ This work does not intend to provide any rigorous quantitative treatment of the chemistry of the system but it only shows the effects induced by the cavity environment.

The torsion of the double bond induces interesting properties in the electronic structure of ethylene, in particular when the dihedral angle approaches 90°. For this particular geometry, the two partially occupied (Frontier) orbitals are practically degenerate and have occupation numbers equal to 1. This configuration confers a high multiconfigurational character to the system. In this section we focus our attention on the effects induced by the field on the Frontier orbitals energies for ethylene in the θ = 90° configuration. The calculations were performed using a cc-pVDZ basis set and a minimal active space containing the two π electrons in the π and π* orbitals, CAS­(2,2), and only one photon was considered. In this case, the cavity frequency has been chosen to match the excitation energy of the lowest bright electronic transition computed at the linear response CASSCF level of theory of the θ = 90° geometry (ω ≈ 3.30 eV). The polarization direction has been directed along the main molecular axis (*x*). The orbital energy profiles were recorded by increasing the light-matter coupling λ from 0 to 0.4 au (see Figure 7). When the coupling is increased, the orbital energies remain almost constant until coupling values of about 0.25 au and then change rapidly, reaching a gap of ∼4 eV (see Figure 7a). This breaking of the orbital degeneracy, with consequent reduction of the multiconfigurational character of the system, reflects also on the orbitals’ occupations (see Figure 7b). Now, one of the Frontier orbitals appears occupied with more than one electron, while the other is occupied with small fractions of an electron. The sudden lifting of the degeneracy of the Frontier orbitals at λ = 0.29 au was further investigated. This behavior can be explained by looking at the energy profiles in Figure 7d where a state inversion happens between λ = 0.29 and 0.35 au. This clearly explains the sudden change in the orbitals’ nature, as observed in Figure 7c. In this case we can observe a field-induced mixing between the involved molecular orbitals. Due to the almost degeneracy of the states, convergence to one or the other can be obtained using different starting guesses.

**Figure 7:**
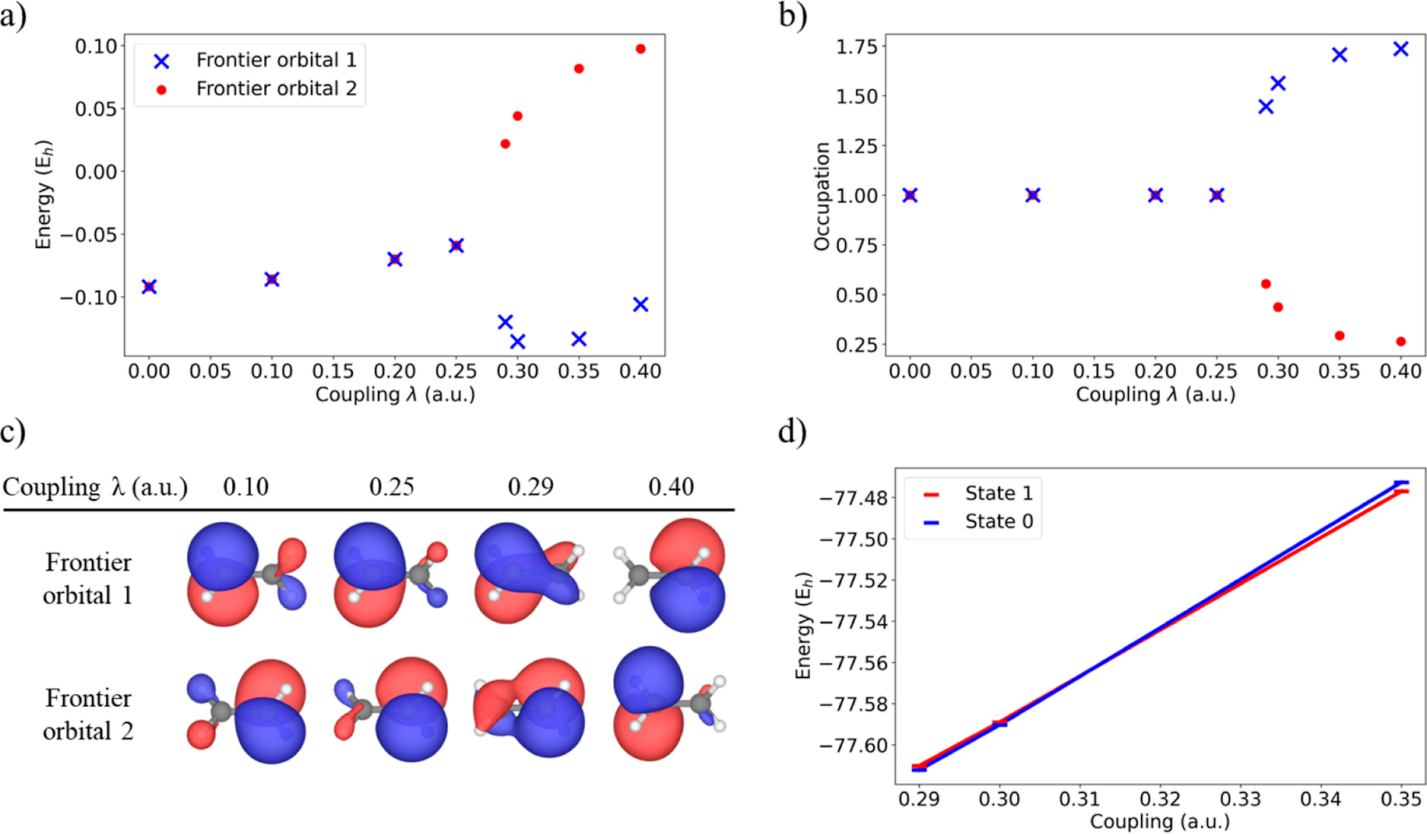
QED-CASSCF Frontier orbitals (a) energies and (b) occupations as a function of the light-matter coupling value. (c) Frontier orbitals’ shapes for different coupling values. (d) Energy profiles of the two states between λ = 0.29 and 0.35 au.

Summarizing, in this paper we observed that in certain conditions the field may induce changes in the multiconfigurational character of a molecular system. The analysis performed in this work has been conducted with light-matter coupling values that are way larger than those currently obtainable in actual experiments. However, if systems with smaller excitation energies are investigated and the method is coupled with strategies to properly account for collective effects, our methodology has the potential to become a reference approach to simulate field-induced effects on the electronic structure of multireference systems.

### 4.3 QED-CASSCF Origin Dependence

It is now well established (as demonstrated in ref 46) that QED-HF theory has an intrinsic origin dependence if charged systems are investigated. This origin dependence does not appear on the total energy because of the origin invariance of Hamiltonian in eq 17 but it is very evident on the molecular orbitals and on their energies. This behavior has been addressed in detail by Riso et al. in ref 104. In particular, they developed an alternative methodology known as strong coupling quantum electrodynamics Hartree–Fock (SC-QED-HF)^104,105^ with the intent of constructing a consistent molecular orbital theory for molecules in strong coupling conditions. In this framework, the wave function is transformed by a unitary transformation, and the orbitals are defined within an electron-photon correlation basis obtained by diagonalizing the operator (**d**·**ϵ**). This approach provides fully origin-invariant molecular orbitals and is able to recover part of electron-photon correlation energy. Therefore, SC-QED-HF is a better fit for the investigation of cavity-induced effects and provides a physically meaningful guess for performing post-HF calculations on charged systems. Recently, the strong coupling formalism has also been extended to second-order Møller–Plesset theory and response theory.^52,106^

Since also CASSCF involves an optimization of the molecular orbitals, it makes sense to check whether the origin invariance problem also affect this method when charged systems are investigated.

In this section we address this point in detail. In particular, we performed a simple test case on the hydroxide ion. Here, CAS­(2,2) calculations were performed while shifting the molecule along the *z* axis (the origin of the reference system has been centered on the oxygen atom). The cavity frequency was set, in this case, to ω = 0.5 eV, the light-matter coupling to λ = 0.03 au, and the polarization vector is oriented along the direction $\varepsilon =\left(\frac{1}{\sqrt{3}},\frac{1}{\sqrt{3}},\frac{1}{\sqrt{3}}\right)$ . We compared the energy of the coherent-state (CS) transformed Hamiltonian (eq 17) to its untransformed version (obtained by setting ⟨d̂⟩=0 ), often known as photon-number (PN) Hamiltonian. Results are presented in Figure 8. In this plot we see that while the total energy computed with the CS-Hamiltonian is origin independent, the results obtained by PN-QED-CASSCF have, as expected, an explicit dependence on the choice of the reference system. This dependence disappears if a large number of photonic states is included into the treatment. However, considering that increasing the dimension of the photonic Fock space results in a higher computational costs, the coherent-state transformation offers a more cost-effective and reliable strategy for the treatment of these systems.

**Figure 8:**
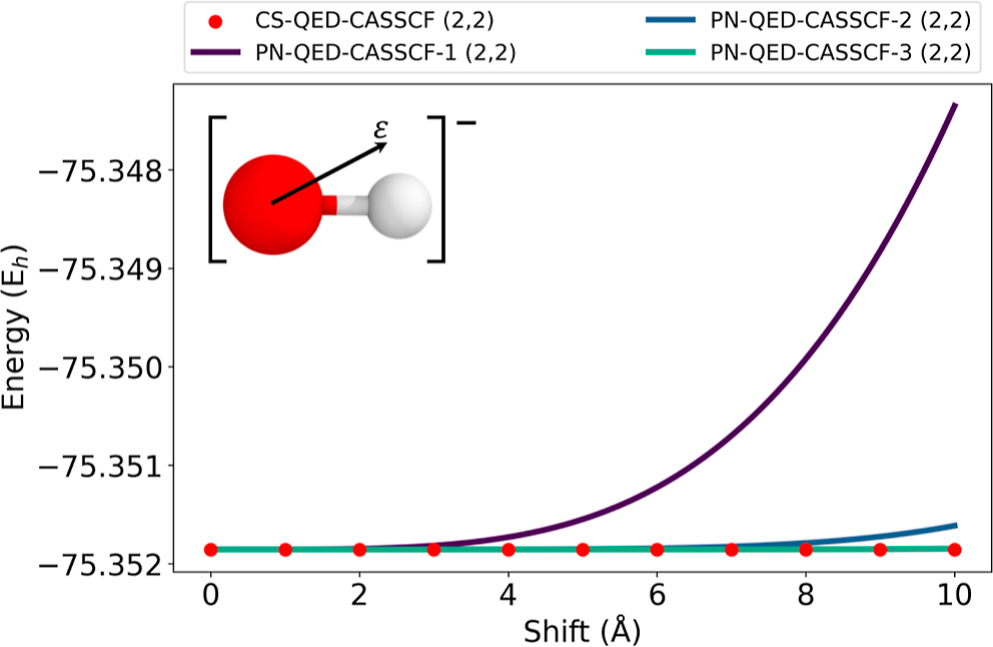
Profile of the QED-CASSCF energy computed with the PN-Hamiltonian and compared with the CS-transformed Hamiltonian for the OH^–^ ion.

It is important to point out that the origin dependence should still be present in the molecular orbitals. This can be expected by the explicit origin dependence of the QED-CASSCF Fock matrix of eq 40. This problem can be eventually fixed by applying a unitary transformation similar to the one used in ref 104 to the CASSCF procedure. However, the application of this transformation to QED-CASSCF goes, for the moment, beyond the scope of this paper.

## 5 Conclusions

In this work we have presented an extension of the complete active space self-consistent field method to account for the effect of quantized electromagnetic fields in optical cavities (QED-CASSCF). The implementation of the method was performed according to a restricted-step second-order optimization method. This strategy allows us to overcome some of the convergence issues that characterize the CASSCF wave function optimization.^67,85^

The developed method was tested on small multireference molecules where the inclusion of static correlation is crucial to provide at least a qualitative description of the system. The comparison between QED-CASSCF and QED-CASCI for the dissociation curve of the nitrogen molecule proved the importance of the orbital optimization to avoid unphysical behaviors arising from possible poor quality guesses.

We also investigated the effects induced by the cavity field on the ethylene torsion process. Our calculations show that the presence of the electromagnetic field can remove the degeneracy between the π and π* orbitals reducing, in specific coupling conditions, the multireference character of the system.

Finally, we addressed the origin dependence of the method in the case of charged systems. In particular, the simple case study of the OH^–^ ion showed that, when the CS-Hamiltonian is used, the total energy is invariant with respect to the choice of origin. In contrast, the photon number PN-Hamiltonian exhibits origin dependence, although the energy converges toward the origin-independent result as the size of the photonic Fock space increases. However, the origin dependence of the molecular orbitals persists even when employing the CS-transformed Hamiltonian. This issue will be solved in a future paper where the Strong Coupling transformation proposed in ref 104 will be applied to remove the origin-dependence of QED-CASSCF also for charged systems.

It is important to remind that, the results obtained in this paper, due to the very large coupling values we used, are not directly comparable with experimental results. However, the aim of the paper, was not much to provide accurate simulations of molecular systems in optical cavities, but more to propose a new methodology able to investigate multireference systems coupled to electromagnetic fields. In the near future this methodology will be coupled with strategies to include collective effects that will allow for more realistic simulations.^107–109^

With this work we move one step further toward an accurate description of multiconfigurational molecular systems in optical cavities. This paves the way for the investigation of chemically/photochemically relevant processes that until this day have been investigated through model Hamiltonians or other approximated methods.^110–112^

## Supplementary Material



## Data Availability

The $e^{T}$ program used to perform the calculations shown in this work is available from the corresponding author upon reasonable request. Examples of the input files used to run the calculations are available at the following Zenodo link: 10.5281/zenodo.15118476.

## Notes

The Supporting Information contains the plot of the QED-CASSCF potential energy curve for the nitrogen molecule computed with an increasing number of photons, the comparison between the CASCI and QED-CASCI curves obtained from independent guesses, and the QED-CASCI curves computed using the high and low energy QED-HF guesses (PDF)
