# Supplementary material for: A Complete Active Space Self-Consistent Field Approach for Molecules in QED Environments
Source: J Chem Theory Comput. 2025 Jul 7;21(14):6862–73. doi: 10.1021/acs.jctc.5c00519 (PMC12288015; doi:10.1021/acs.jctc.5c00519)
Supplement: Supplementary file 1 [file ct5c00519_si_001.pdf]

Supporting Information: A Complete Active Space Self-Consistent Field approach for molecules in QED environments

Riccardo Alessandro,† Matteo Castagnola,‡ Henrik Koch,‡ and Enrico Ronca*,†

†*Dipartimento di Chimica, Biologia e Biotecnologie, Università degli Studi di Perugia, Via Elce di Sotto, 8,06123, Perugia, Italy*

‡*Department of Chemistry, Norwegian University of Science and Technology, 7491 Trondheim, Norway*

E-mail: enrico.ronca@unipg.it

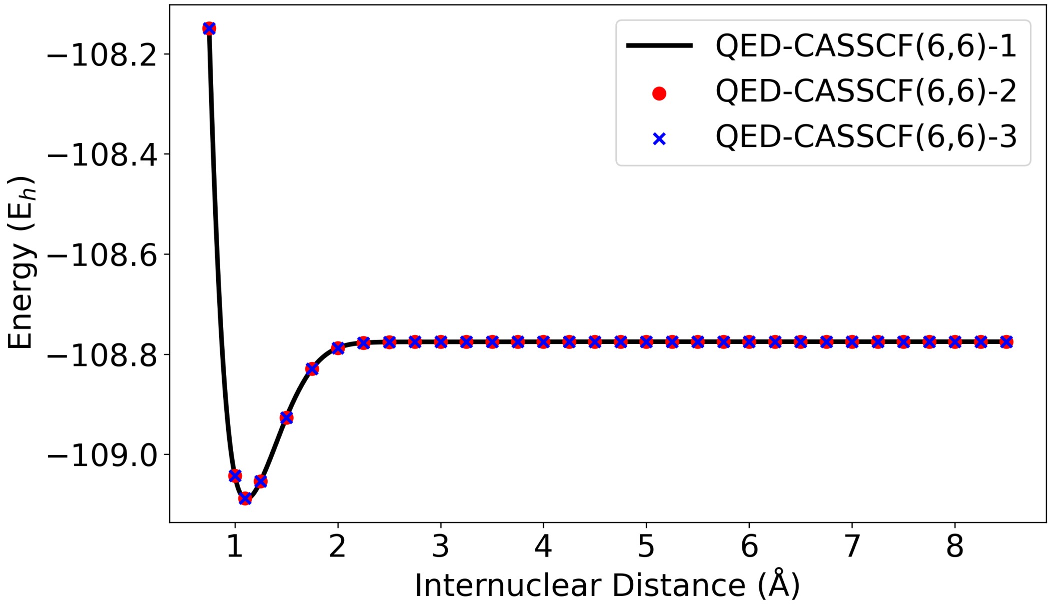

Figure S1. Potential energy curve for the nitrogen molecule computed at the QED-CASSCF level with an increasing number of photons.

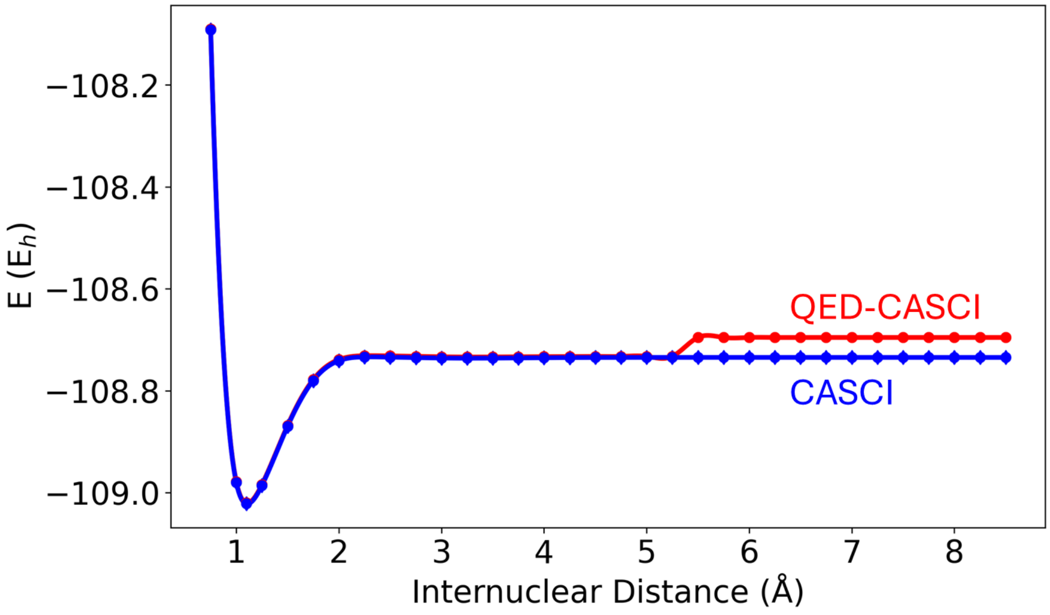

Figure S2. Comparison between the CASCI(6,6) and QED-CASCI(6,6) potential energy curves for the nitrogen molecule.

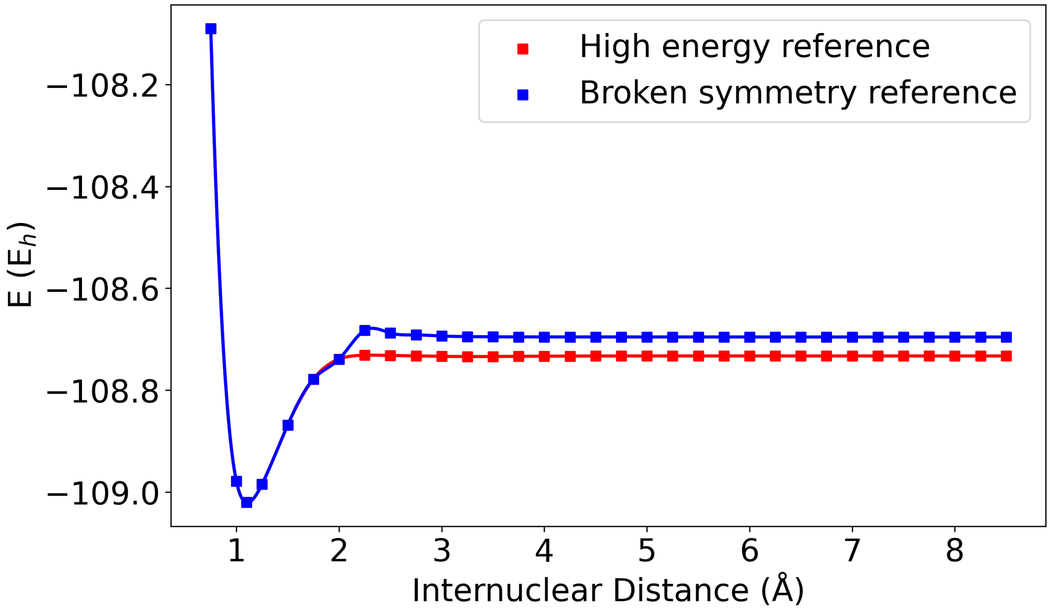

Figure S3. QED-CASCI(6,6) curves obtained for the nitrogen molecule by using the high energy and broken symmetry orbitals as a guess.

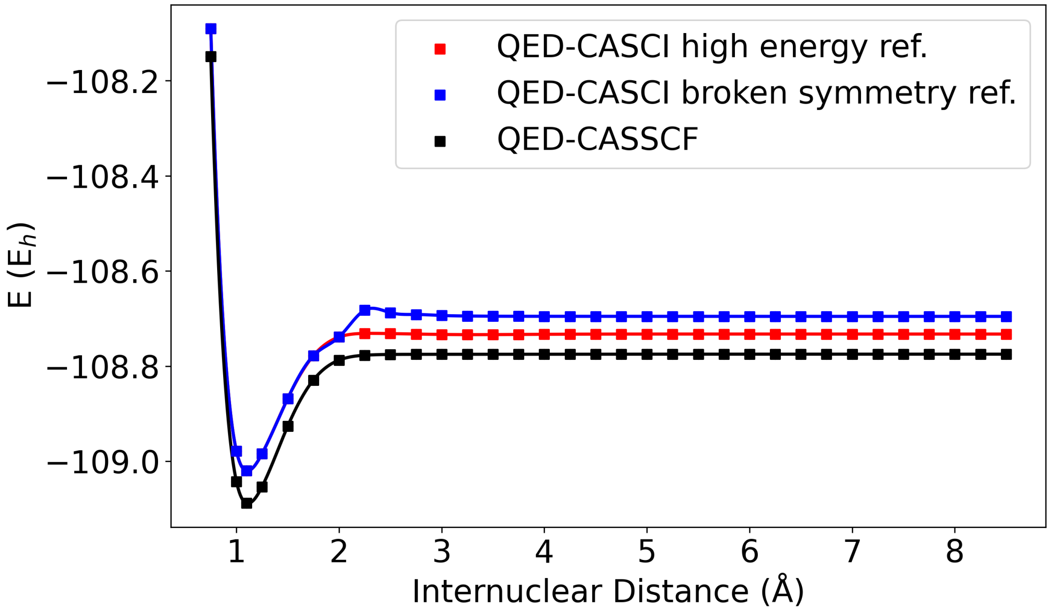

Figure S4. Comparison between the potential energy curves for the nitrogen molecule at the QED-CASCI(6,6) level obtained by using the high energy and broken symmetry orbitals as a guess and the QED-CASSCF(6,6) curve.
